# Potential therapeutic strategies for quercetin targeting critical pathological mechanisms associated with colon adenocarcinoma and COVID-19

**DOI:** 10.3389/fphar.2022.988153

**Published:** 2022-09-29

**Authors:** Xiushen Li, Weizheng Liang, Chengwei Yu, Qingxue Meng, Weiwen Zhang, Xueliang Wu, Jun Xue, Shoulong Deng, Hao Wang

**Affiliations:** ^1^ Department of Obstetrics and Gynecology, Shenzhen University General Hospital, Shenzhen, China; ^2^ Guangdong Key Laboratory for Biomedical Measurements and Ultrasound Imaging, School of Biomedical Engineering, Shenzhen University Health Science Center, Shenzhen, China; ^3^ Shenzhen Key Laboratory, Shenzhen University General Hospital, Shenzhen, China; ^4^ Central Laboratory, The First Affiliated Hospital of Hebei North University, Zhangjiakou, China; ^5^ School of Future Technology, University of Chinese Academy of Sciences, Beijing, China; ^6^ Department of General Surgery, The First Affiliated Hospital of Hebei North University, Zhangjiakou, China; ^7^ National Health Commission of China (NHC) Key Laboratory of Human Disease Comparative Medicine, Institute of Laboratory Animal Sciences, Chinese Academy of Medical Sciences and Comparative Medicine Center, Peking Union Medical College, Beijing, China

**Keywords:** quercetin, colon adenocarcinoma, COVID-19, prognostic model, bioinformatics, network pharmacology

## Abstract

Patients with colon adenocarcinoma (COAD) are at a higher probability of infection with COVID-19 than healthy individuals. However, there is no globally accepted treatment protocol for patients with COAD/COVID-19. Quercetin has been found to have significant antitumor, antiviral and anti-inflammatory effects in several studies. Therefore, this study sought to evaluate the potential of quercetin as the agent for COAD/COVID-19 and to explore its mechanisms. We used bioinformatics algorithms to obtain COAD/COVID-19-related genes (CCRG) from COAD-related transcriptome data and COVID-related transcriptome sequencing data, and used these genes to construct a COAD prognostic model. We intersected the CCRG with the therapeutic target genes of quercetin and obtained a total of 105 genes (potential target genes of quercetin for the treatment of COAD/COVID-19). By constructing a protein-protein interaction (PPI) network, we ascertained FOS, NFKB1, NFKB1A, JUNB, and JUN as possible core target genes of quercetin for the treatment of COAD/COVID-19. Bioinformatic analysis of these 105 genes revealed that the mechanisms for quercetin the treatment of COAD/COVID-19 may be associated with oxidative stress, apoptosis, anti-inflammatory, immune, anti-viral and multiple pathways containing IL-17, TNF, HIF-1. In this study, we constructed a prognostic model of COAD/COVID19 patients by using CCRG and elucidated for the first time the potential target genes and molecular mechanisms of quercetin for the treatment of COAD/COVID-19, which may benefit the clinical treatment of COAD/COVID-19 patients. However, no clinical trials have yet been conducted to further validate the findings, but this will be the future direction of our research.

## Introduction

Since the emergence of the SARS-CoV-2 virus in 2019, it continues to have an immeasurable impact on the lives, health and work of people around the world. Similar to other highly pathogenic coronaviruses, the main modes of transmission of SARS-CoV-2 are close contact, droplets, pollutants, aerosols (Guarner, 2020; Chen et al., 2021). To date, the morbidity and mortality of COVID-19 remains at a high level worldwide (Cetin et al., 2022). According to the World Health Organization on June 28, 2022, the cumulative number of people infected with COVID-19 worldwide has exceeded 540 million and the cases of deaths related to COVID-19 has exceeded 6.3 million (https://covid19.who.int/). The global epidemiological situation is currently improving, but there are still about 300,000 new confirmed COVID-19 patients every day. While significant advances have been made in COVID-19-related assays and vaccine, there have been no major breakthroughs in COVID-19-related drugs, making the screening of drugs for the clinical treatment of COVID-19 patients become urgent (Li R. et al., 2021; Fenollar et al., 2021; Fiolet et al., 2022).

As the most common gastrointestinal tumor, colon adenocarcinoma (COAD) is a malignant neoplasm that seriously threatens human life. Globally COAD has the fourth highest incidence and second death rates of all oncological diseases (Dekker et al., 2019). In the last decade, great progress has been made in biological research, early screening and clinical treatment of COAD, but the prognosis of COAD patients has not improved markedly (Durinikova et al., 2021). There is an association between cancer and COVID-19, with cancer patients having a higher probability of contracting SARS-CoV-2 and experiencing exacerbations compared to healthy individuals (Han et al., 2021). By collecting and collating examination reports from COVID-19 positive patients, Karla et al. found that cancer patients were 1.6 times more at risk of being infected by SARS-CoV-2 than healthy people, while cancer patients undergoing chemotherapy or immunotherapy were at an even higher risk of infection, approximately 2.2 times (Lee et al., 2021). In addition, SARS-CoV-2 virus contributes to the poor prognosis of COAD patients by affecting the process of immune cell infiltration (Ahmadi et al., 2021). Therefore, there is an urgent need to explore drugs for the treatment of COAD/COVID-19.

As an important part of the world’s traditional medicine, Chinese medicine has a history of over 2000 years. With the application of evidence-based medicine and the development of Chinese medicine research, the clinical efficacy of Chinese medicine has been recognized by mainstream medicine (Lu et al., 2020). Chinese herbs are crucial in the struggle against the COVID-19 outbreak. Quercetin, a type of flavonoid, is found in many herbs, as well as in numerous fruits and vegetables (Reyes-Farias and Carrasco-Pozo, 2019). Research has found that quercetin has antioxidant, anticancer, antiviral and anti-inflammatory effects (Li et al., 2016; Tang et al., 2020; Gansukh et al., 2021; Di Petrillo et al., 2022). MEK1 is the star molecule in the oncogenic RAS signaling pathway and quercetin has been shown to act as an inhibitor of MEK1 with superior inhibition to PD098059, which is a conventional inhibitor of MEK1 (Lee et al., 2008). Quercetin is the natural product that helps to enhance the body’s immune function, prevent viral invasion and suppress the appearance of severe inflammatory responses, thus providing assistance to COVID-19 (Mrityunjaya et al., 2020). Therefore, we used bioinformatics algorithms to systematically assess the potential therapeutic value of quercetin for the COAD/COVID-19 and to explore its molecular mechanisms.

## Materials and methods

### Flowchart

**FIGURE 1:**
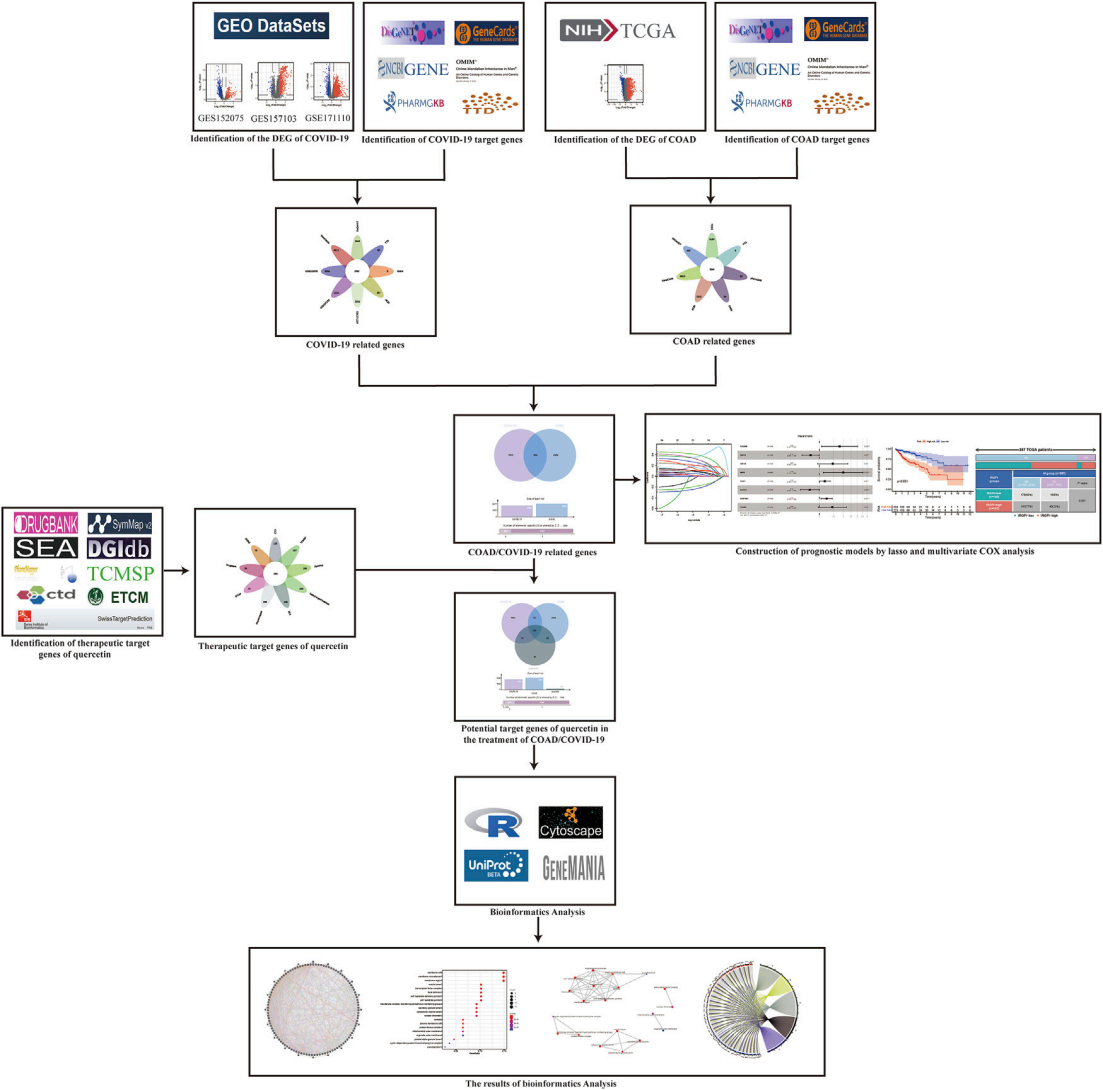
Flow chart.

First, COVID-19 related genes (RG) were identified by using the Gene Expression Omnibus (GEO) database and 6 recognized databases. Then COAD RG were identified by using The Cancer Genome Atlas (TCGA) database and 6 recognized databases. The intersection of COAD RG and COVID-19 RG was used to construct a COAD prognostic model and to further evaluate the predictive power. Next, the therapeutic target genes of quercetin were queried through 9 drug target databases. The therapeutic target genes of quercetin, COVID RG and COAD RG were intersected. Finally, the molecular mechanism of quercetin as a therapeutic agent for patients with COVID-19/COAD was explored by bioinformatics algorithms (Figure 1).

### Identification of COVID-19 RG and COAD RG

3 COVID-19 related datasets, GSE157103, GSE152075 and GSE171110, were obtained though the GEO database. By the “limma " package in R (filtered by *p* < 0.05 and | log fold change | >1), we screened differentially expressed genes (DEG) from these 3 datasets for COVID-19 patients, respectively. Next, we identified COVID-19 target genes in the DisGeNET, TTD, GeneCards, OMIM, NCBI and pharmaGKB databases by using the keyword " COVID-19". Genes with greater than or equal to 2 occurrences were considered to be COVID-19 RG.

We used the same method and filtering criteria to extract the DEG of COAD from the TCGA-COAD dataset. 6 databases, including DisGeNET, GeneCards, NCBI, OMIM, pharmaGKB, and TTD, were then employed to retrieve the target genes for COAD by using the keyword “colon adenocarcinoma”. Genes with greater than or equal to 2 occurrences were considered to be COVID RG.

### Construction of COAD prognostic model

The COAD prognostic model was constructed according to the following steps: 1) Perform the univariate Cox survival analysis on CCRG by the “survdiff” function and “coxph” function in R to screen for genes associated with prognosis in COAD patients (*p* < 0.05). 2) Use least absolute shrinkage and selection operator (LASSO) Cox regression analysis on genes screened in step 1 by the “glmnet” package in R for downscaling and gene screening. 3) Perform the multivariate Cox survival analysis on genes screened in step 2. 4) Calculate the risk scores for COAD patients based on the results of the analysis in step 3. 5) Divide into low- and high-risk groups based on the risk scores of COAD patients. 6) Construct the nomogram prediction models by using risk scores and clinical characteristics.

### Evaluation of the COAD prognostic model

The steps for evaluating the prognostic model for COAD were as follows: 1) Observe predictive effect of the prediction model by Kaplan–Meier (KM) survival curves. 2) Calculate area under the curve (AUC) of the prediction model by constructing receiver operating characteristic (ROC) curves by the “timeROC” package in R. 3) Use ROC curves and concordance index (C-index) to compare predictive performance of model with the genes used to construct the model and clinical traits.

### Identification of quercetin therapeutic target genes

To obtain the target genes of quercetin, we searched 9 databases: 1) CTD; 2) DGIdb; 3) DrugBank; 4) ECTM; 5) PharmMapper; 6) SEA; 7) SwissTargetPrediction; 8) SymMap; 9) TCMSP. Genes that appeared in 2 or more databases were considered to be therapeutic target genes for quercetin.

### Bioinformatics analysis

The intersection of quercetin treatment target genes, CCRG was uploaded to the GeneMANIA website for the construction of protein-protein interaction (PPI) network. The data from PPI network were loaded into Cytoscape software to screen the core target genes of quercetin for COAD/COVID-19 by the degree values.

Gene ontology (GO) and Kyoto Encyclopedia of Genes and Genomes (KEGG) enrichment analysis of the CCRG was performed though the “clusterProfiler” package in R. The “ggplot2” package and the “circlize” package in R were used to visualize the results.

## Results

### Identification of COAD RG and COVID-19 RG

**FIGURE 2:**
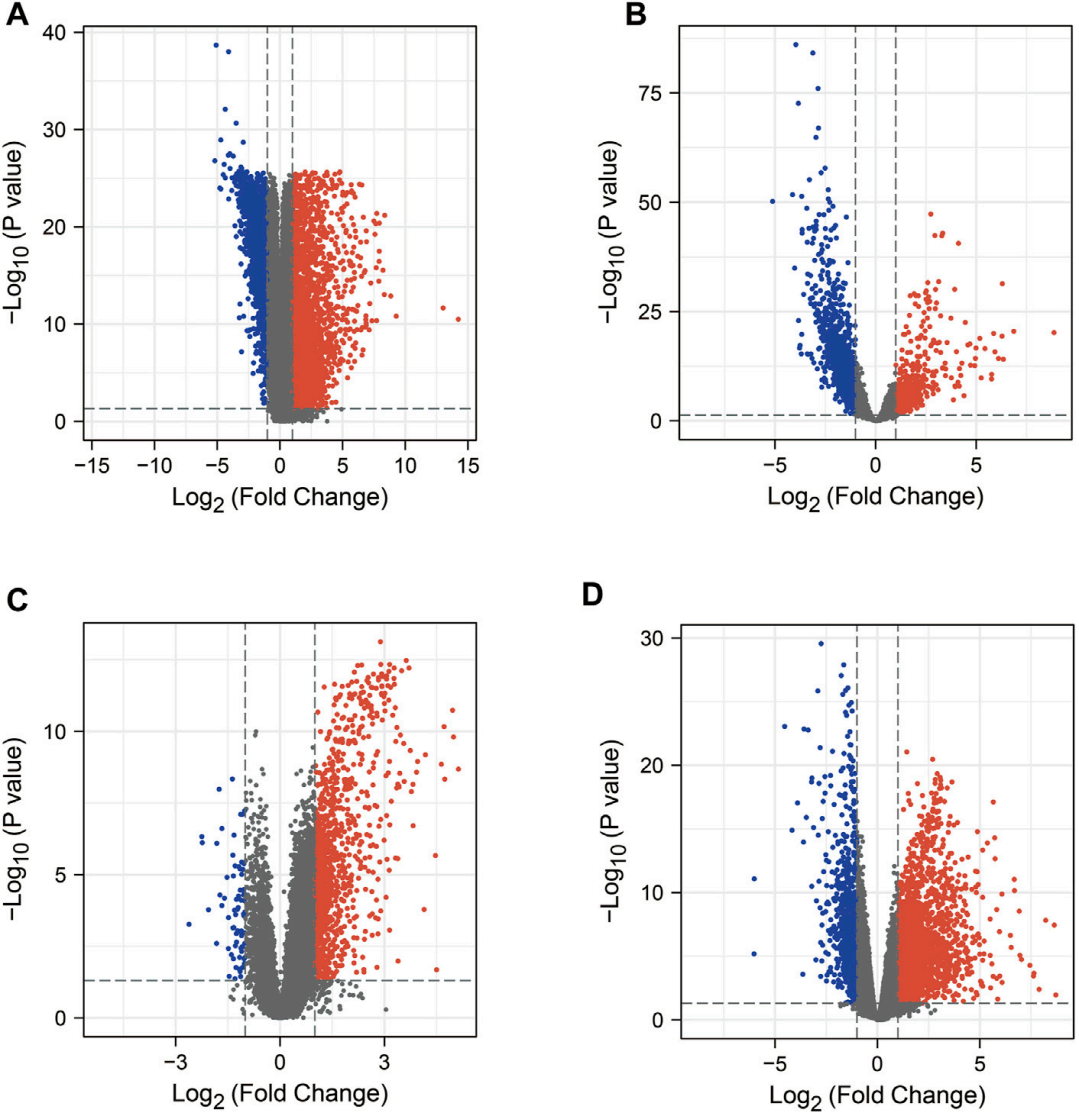
Volcano plots of mRNA expression in COAD or COVID-19 patients versus healthy individuals, respectively. **(A)** Volcano plot of the TCGA-COAD dataset. **(B–D)** Volcano plots of DEG in the GSE152075 dataset, GSE157103 dataset and GSE171110 dataset. COAD: colon adenocarcinoma; DEG: differentially expressed gene.

**FIGURE 3:**
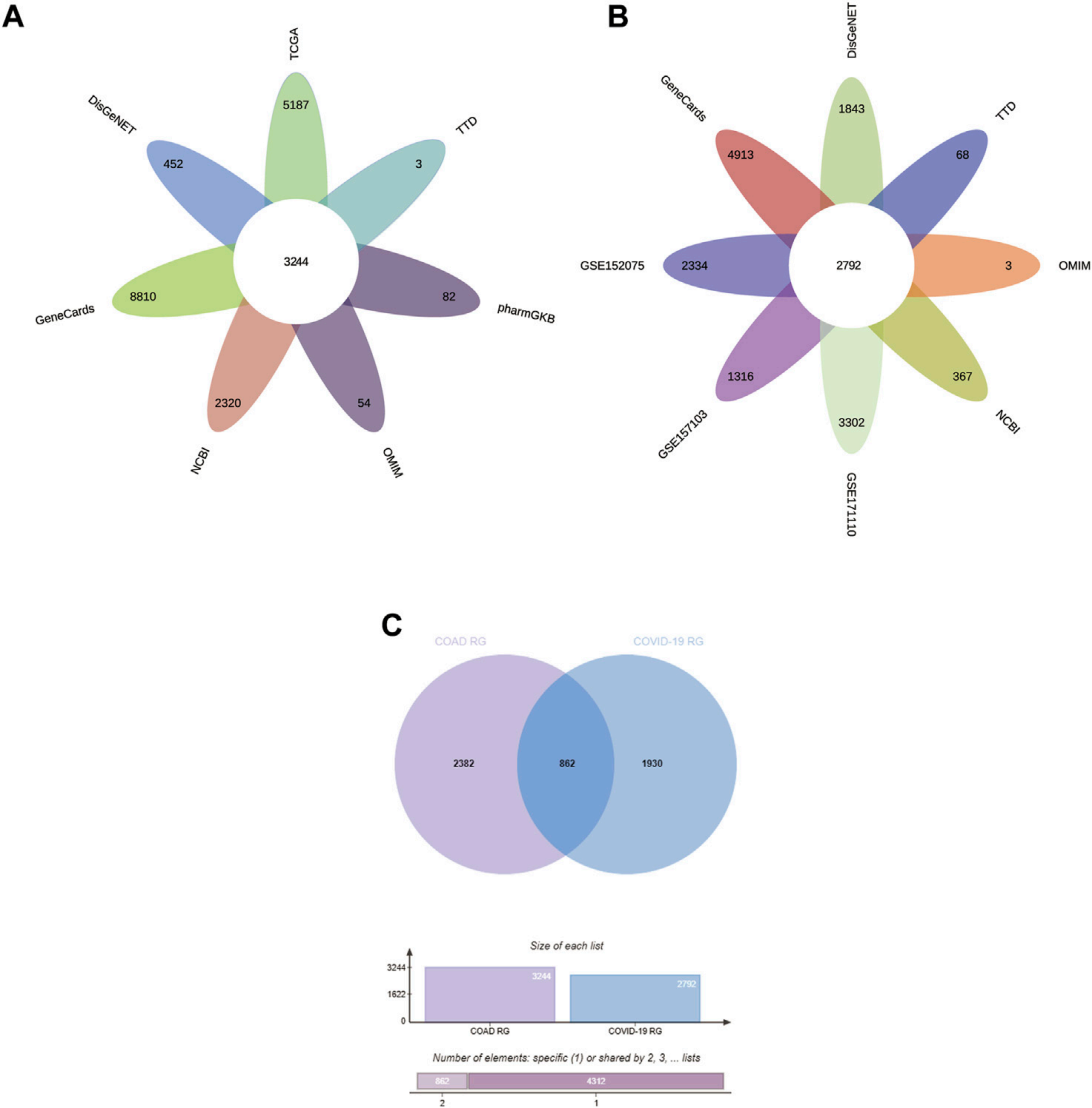
Identification of CCRG. **(A–C)** Venn diagram of COAD RG, COVID-19 RG, and CCRG. CCRG: COAD/COVID-19 related genes; RG: related genes.

In this study, we obtained 5187 DEG from the COAD dataset (containing transcriptomic data from 471 COAD tissues and 41 normal tissues) in the TCGA database by the “limma” package in R (Figure 2A). In addition, searches of the DisGeNET, GeneCards, NCBI, OMIM, pharmaGKB, and TTD databases yielded 452, 8810, 2320, 54, 82, and 3 target genes associated with COAD, respectively. To increase the accuracy of this study, we only referred to genes with ≥2 occurrences as COAD RG. As shown in Figure 3A, we obtained a total of 3244 COAD RG.

We explored the DEG in COVID-19 patients in the 3 GEO datasets by the “limma” package in R. In the GSE152075 dataset (containing transcriptomic data from nasopharyngeal swabs of 430 COVID-19 patients and 54 healthy individuals), 2334 DEGs were obtained (Figure 2B). In the dataset GSE157103 (containing transcriptomic data from plasma samples of 100 COVID-19 patients and 26 healthy individuals), 1316 DEG were obtained (Figure 2C). In the GSE171110 dataset (containing transcriptomic data from whole blood samples of 44 COVID-19 patients and 10 healthy individuals), 3302 DEG were obtained (Figure 2D). In addition, searches of the DisGeNET, GeneCards, NCBI, OMIM, pharmaGKB and TTD databases yielded 1843, 4913, 367, 3, 0 and 68 target genes associated with COVID-19, respectively. Genes with ≥2 occurrences were referred as COVID-19 RG. Ultimately, we obtained 2792 COVID-19 RG (Figure 3D).

We performed the operation of taking the intersection of the obtained COAD RG and COVID-19 RG and obtained 862 CCRG (Figure 3C).

### Construction and evaluation of the COAD prognostic model

**FIGURE 4:**
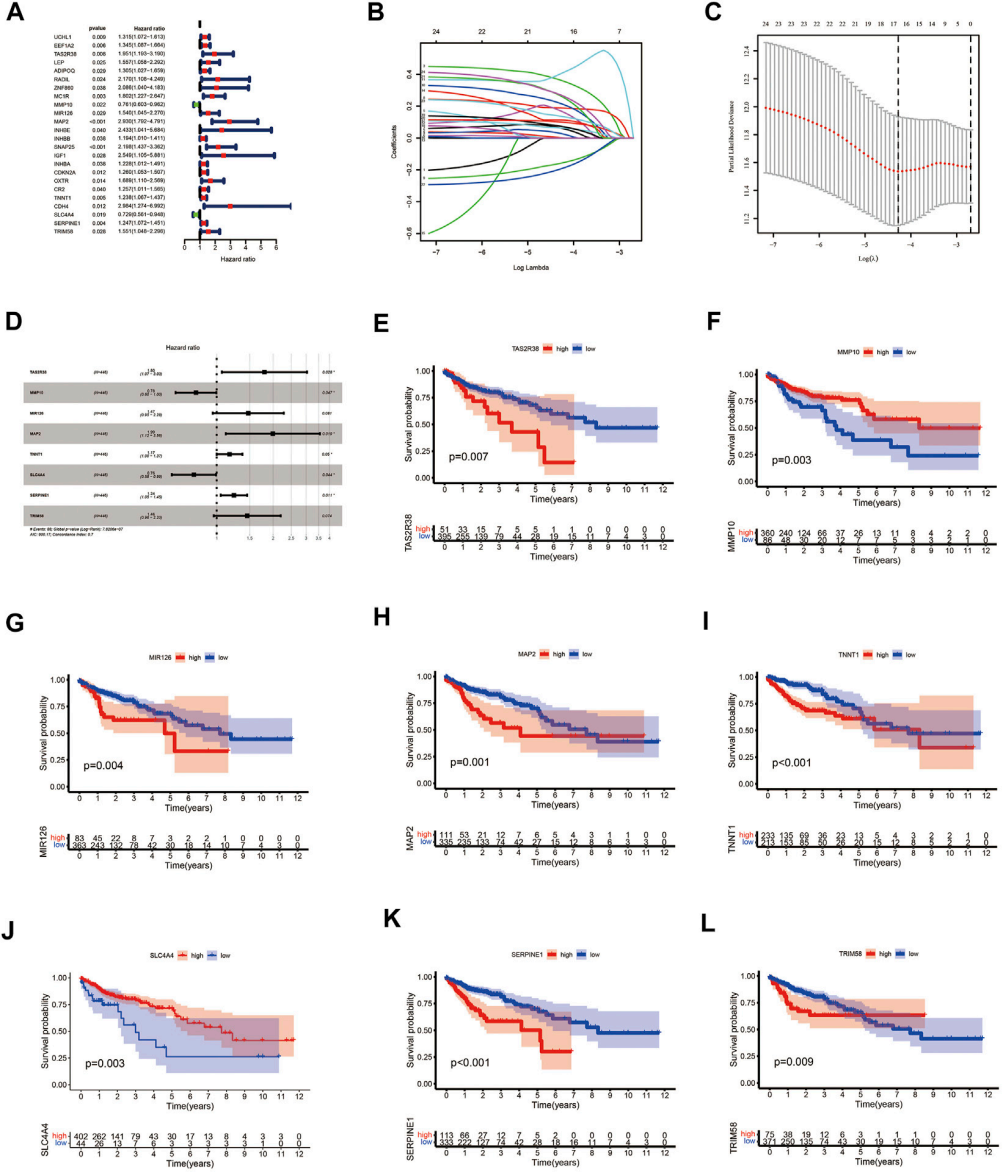
Prognostic model of COAD patients constructed by using CCRG. **(A)** Forest plots of univariate Cox survival analysis of CCRG. **(B, C)** Coefficient profiles and cross-validation error rate plots for LASSO regression analysis of CCRG obtained from outcomes of univariate Cox survival analysis, respectively. **(D)** Forest plots of multivariate Cox survival analysis of genes obtained from outcomes of LASSO regression analysis. **(E–L)** KM survival curves of genes obtained from outcomes of multivariate Cox analysis. COAD: colon adenocarcinoma; CCRG: COAD/COVID-19 related genes; KM: Kaplan–Meier.

**FIGURE 5:**
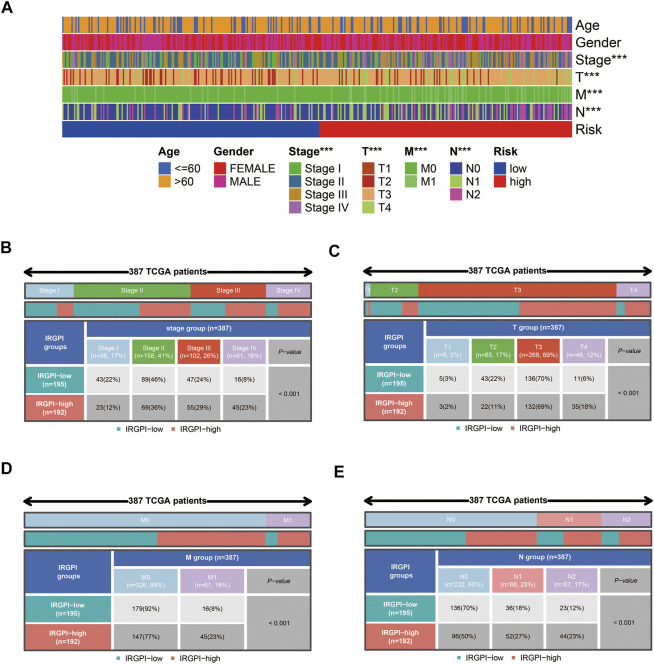
Clinicopathological information for COAD patients in low- and high-risk groups. **(A)** Distribution of clinicopathological characteristics of patients (***p < 0.001). **(B–E)** Distribution of patients with different Stages, T, M and N. COAD: colon adenocarcinoma.

We performed univariate Cox survival analysis on CCRG and obtained a total of 24 RG with prognosis in patients with CAOD (*p* < 0.05) (Figure 4A). Next, these 24 RG were downscaled by LASSO regression analysis, leaving 14 genes for the next analysis (Figures 4B,C). Finally, we performed multivariate Cox survival analysis on these 14 RG and screened out 8 genes containing TAS2R38, MMP10, MIR126, MAP2, TNNT1, SLC4A4, SERPINE1 and TRIM58 for the construction of prognostic model (Figure 4D). Among them, MMP10 and SLC4A4 were protective factors, while TAS2R38, MIR126, MAP2, TNNT1, SERPINE1 and TRIM58 were risk factors. By plotting KM survival curves for these 8 RG, we found high expression of MMP10 and SLC4A4 was associated with good prognosis, while high expression of TAS2R38, MIR126, MAP2, TNNT1, SERPINE1 and TRIM58 were associated with poor prognosis, in line with the results of multivariable Cox survival analysis (Figures 4E–4L). Further analysis of the distribution of patients’ clinical traits in low- and high-risk groups (Figure 5A), we found that patients’ clinical traits containing Stage, T, M and N were associated with risk scores (*p* < 0.001) (Figures 5B–E).

### Evaluation of the COAD prognostic model

**FIGURE 6:**
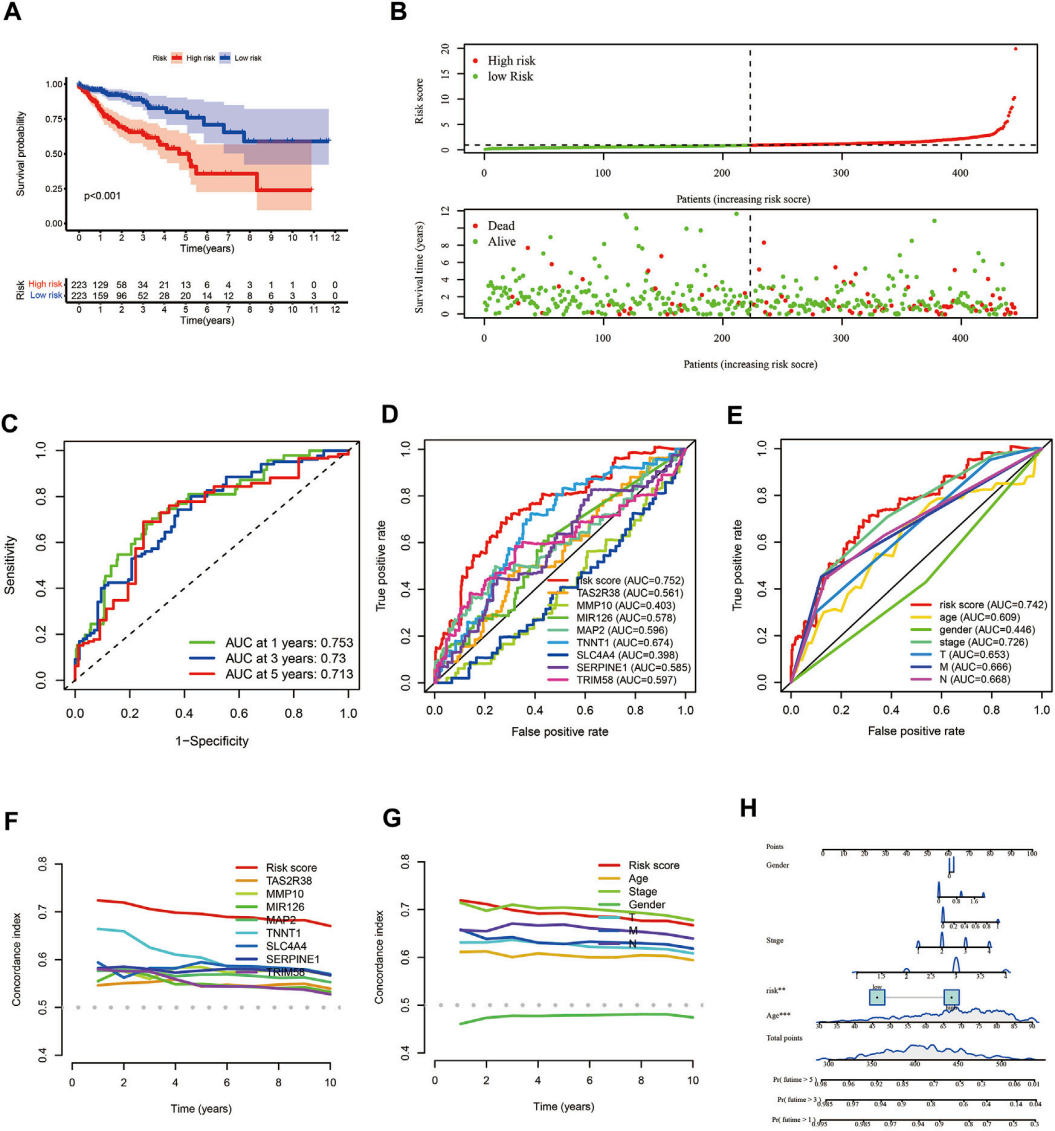
Evaluation of prognostic models for COAD patients. **(A)** KM survival analysis. **(B)** Risk scores and survival status of COAD patients. **(C)** ROC curves of 1, 3 and 5 years. **(D, F)** ROC curves and C-index plots for the model and the genes used to construct the model, respectively. **(E, G)** ROC curves and C-index plots of the model and the patient’s clinical information, respectively. **(H)** The nomogram prediction model constructed by using risk score and clinical information of COAD patients. COAD: colon adenocarcinoma; KM: Kaplan–Meier; ROC, receiver operating characteristic; C-index: concordance index.

The results of the survival analysis found that COAD patients in the low-risk group had a significantly longer survival time than those in the high-risk group (*p* < 0.001) (Figure 6A). As the risk score increased, the number of COAD patients who died increased (Figure 6B). ROC curves were plotted to assess the accuracy of the model constructed in this study. The results showed that the model had good predictive performance with AUC of 0.753, 0.73 and 0.713 for 1, 3 and 5 years respectively (Figure 6C). We then compared the predictive efficiency of the model with that of the genes for which it was constructed, and the results of the ROC curve and concordance index (C-index) showed that the model had better predictive efficacy (Figures 6D,F). The ROC curves for the model and the patient’s clinical traits also showed that the model had good predictive power, with the C-index being only lower than the patient’s stage (Figures 6E,G). Finally, we combined the model with the patient’s clinical traits to construct the nomogram that could be used to predict the patient’s prognosis (Figure 6H).

### Identification of therapeutic target genes of quercetin

**FIGURE 7:**
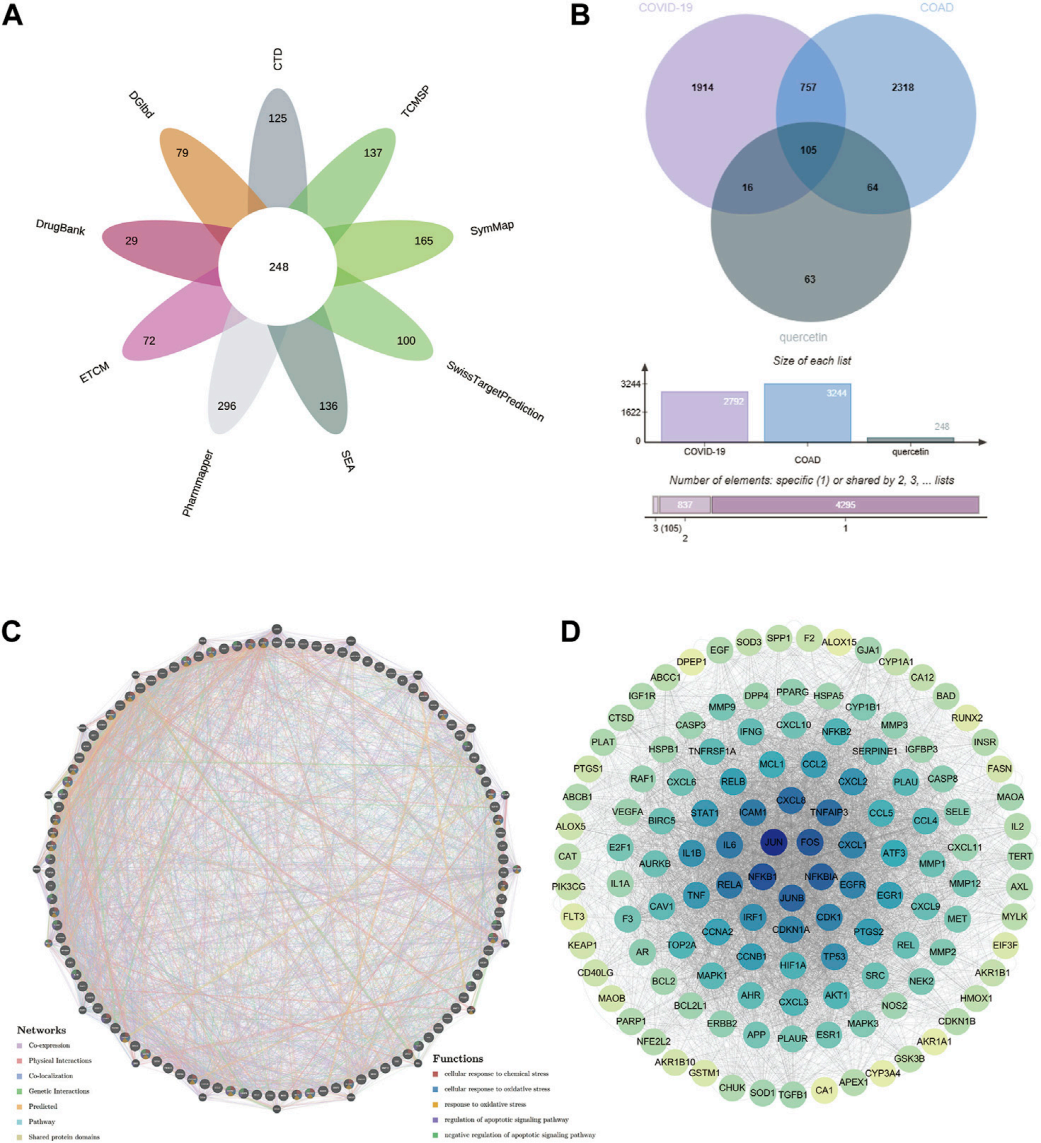
Potential target genes of quercetin for the treatment of COAD/COVID-19 and the corresponding PPI network. **(A)** Target genes of quercetin from 9 databases. **(B)** Potential target genes of quercetin for the treatment COAD/COVID-19. **(C)** PPI network of potential target genes. **(D)** Degree map of the PPI network. COAD: colon adenocarcinoma; PPI: protein-protein interaction.

By using “quercetin” as the keyword, we searched 9 databases including CTD, DGIdb, DrugBank, ECTM, PharmMapper, SEA, SwissTargetPrediction, SymMap, TCMSP, and obtained 125, 79, 29, 72, 296, 136, 100, 165 and 137 quercetin target genes. As shown in Figure 7A, we obtained a sum of 248 quercetin therapeutic target genes by using the number of gene occurrences ≥2 as the screening condition.

### Potential target genes of quercetin for the treatment of COAD/COVID-19

Taking the intersection of CCRG and target genes of quercetin, we obtained 105 potential target genes of quercetin for the treatment of COAD/COVID-19 (Figure 7B), which we referred to as gene set 1 and used for bioinformatics analysis.

### Construction of the PPI network

GeneMANIA database, based on the label propagation algorithm and linear regression-based algorithm, is used to predict the function of genes and the interactions between genes. We used GeneMANIA to construct a PPI network for gene set 1 (Figure 7C). The circular nodes represented genes (different colors represented different gene enrichment results), while the lines represented the interactions between genes (different colored lines represented different interactions). To find the core target genes of quercetin for the treatment of COAD/COVID-19, we loaded the PPI network data into Cytoscape software and calculated the degree value of each gene (the depth of the color is proportional to the degree value). The mechanism of quercetin for the treatment of COAD/COVID-19 might be related to apoptosis and oxidative stress. FOS, JUN, NFKB1, JUNB, and NFKB1A had the highest degree values (Figure 7D), so we concluded that these genes might be the core target genes of quercetin for the treatment of COAD/COVID-19.

### Results of GO and KEGG enrichment analysis

**FIGURE 8:**
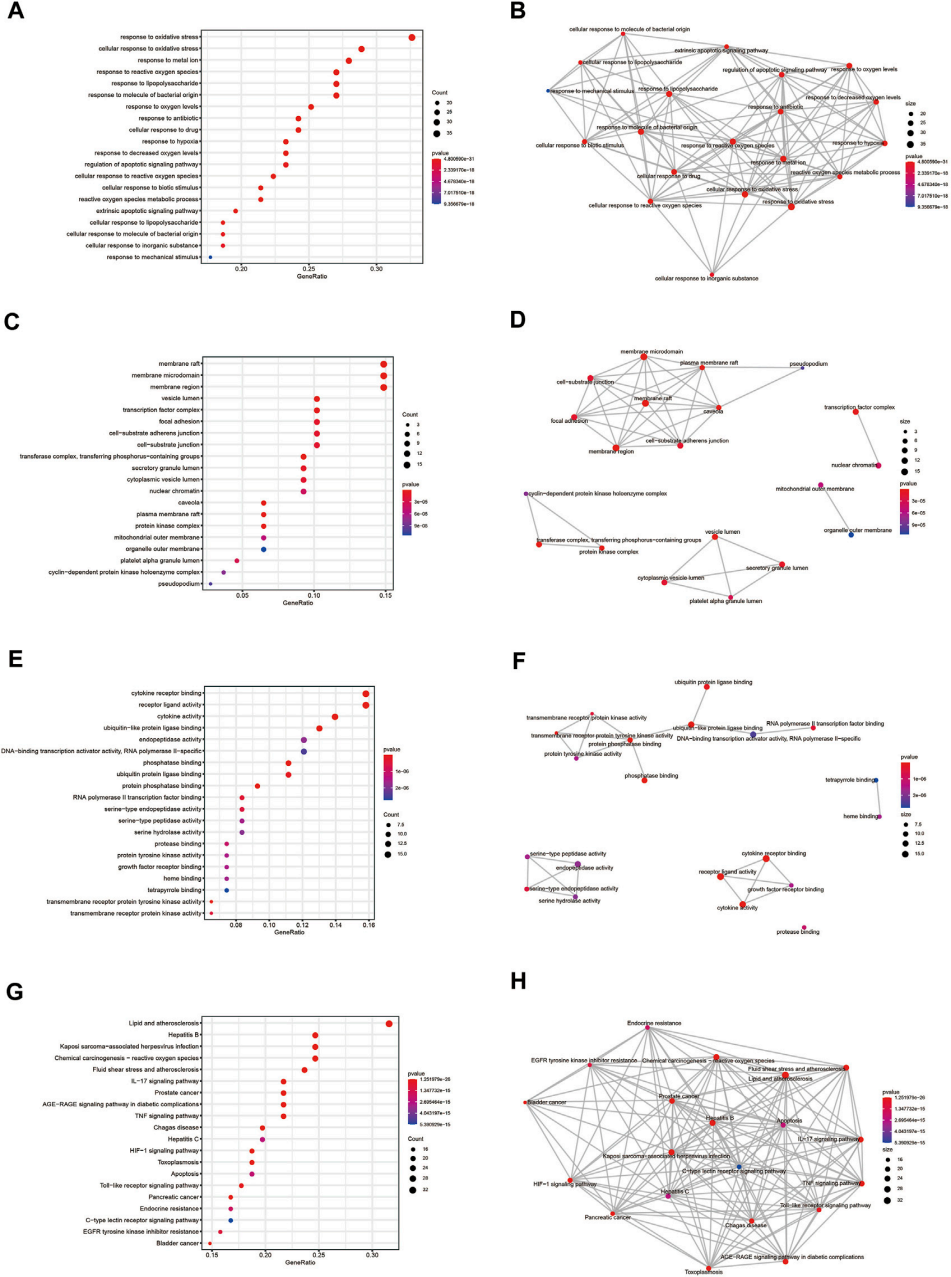
GO and KEGG enrichment analysis. **(A, B)** Outcomes of BP enrichment analysis and correlation analysis. **(C, D)** Outcomes of CC enrichment analysis and correlation analysis. **(E, F)** Outcomes of MF enrichment analysis and correlation analysis. **(G, H)** Outcomes of KEGG enrichment analysis and correlation analysis. GO: Gene ontology; KEGG: Kyoto Encyclopedia of Genes and Genomes; BP: biological processes; CC: cellular components; MF: molecular functions.

**FIGURE 9:**
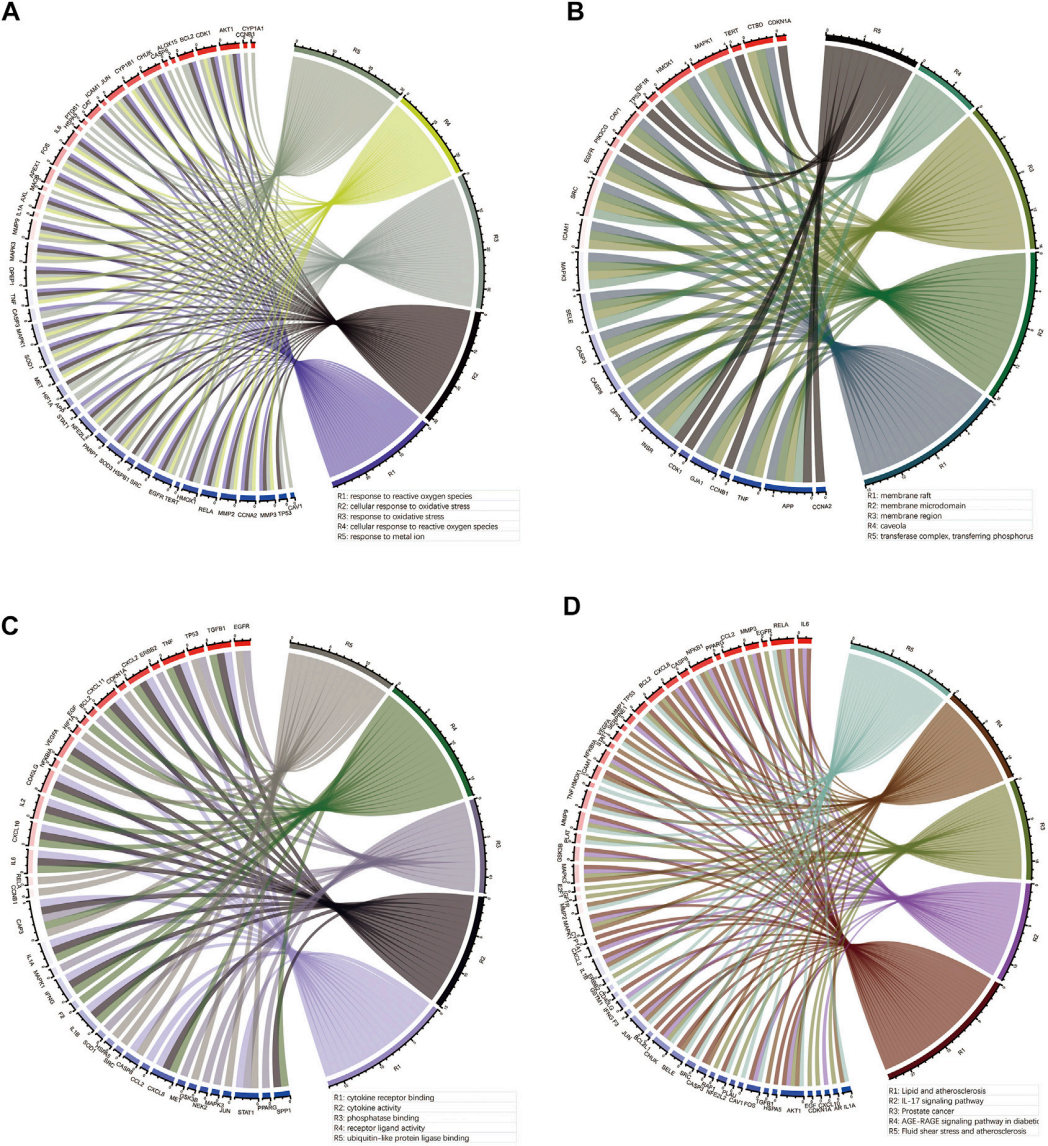
Outcomes of the top 5 bioinformatics analyses and the corresponding genes. **(A–C)** Outcomes of GO enrichment analysis for BP, CC and MF. **(D)** Outcomes of KEGG enrichment analysis. GO: Gene ontology; KEGG: Kyoto Encyclopedia of Genes and Genomes; BP: biological processes; CC: cellular components; MF: molecular functions.

To elucidate the molecular mechanism of quercetin for the treatment of COAD/COVID-19, we performed GO and KEGG enrichment analysis of gene set 1 by the “clusterProfiler” package in R. GO enrichment analysis of biological processes (BP), cellular components (CC), and molecular functions (MF) yielded 2389, 78, and 177 terms, respectively (Supplementary Table S1). We filtered out only the 20 terms for visualization (Figures 8A,C,E) and performed correlation analysis (Figures 8B,D,F). The results of BP enrichment analysis included response to reactive oxygen species, response to oxidative stress, regulation of apoptotic signaling pathway, cellular response to drug, response to oxygen levels, response to molecule of bacterial, cellular response to biotic stimulus etc. The results of CC enrichment analysis included membrane raft, membrane region, caveola, protein kinase complex, plasma membrane adherens junction, antibiotic etc. The results of MF enrichment analysis included cytokine receptor binding, cytokine activity, receptor ligand activity, ubiquitin-like protein ligase binding, protein phosphatase binding, transmembrane receptor protein tyrosine kinase activity, RNA polymerase II transcription factor binding etc. Figures 9A–C showed the 5 terms with the lowest *p*-values and the corresponding enriched genes from the GO enrichment analysis.

Similarly, we performed KEGG enrichment analysis on 105 genes in gene set 1 (Supplementary Table S2). Figures 8G,H showed the 20 KEGG enrichment results with the smallest *p*-values and the correlation analysis between these enrichment results, respectively. The results of KEGG enrichment analysis mainly included Apoptosis, Endocrine resistance and multiple pathways containing IL-17, TNF, HIF-1. Figure 9D showed the 5 terms with the lowest *p*-values and the corresponding enriched genes from the KEGG enrichment analysis.

## Discussion

SARS-CoV-2, a highly infectious and dangerous virus, has caused an unprecedented public crisis worldwide and poses a serious threat to people’s daily lives (Yang and Rao, 2021). Although the widespread availability of the COVID-19 vaccine has slowed the spread of the virus, the number of new COVID-19 patients still does not show any significant downward trend. We have not yet developed a specific drug to treat COVID-19. Chinese medicine has an effective and lasting function in the fight against COVID-19 by modulating excessive inflammatory and immune responses, reducing the risk of developing severe disease and improving patients’ clinical symptoms (Liu et al., 2020; Lyu et al., 2021). The Chinese Drug Administration approved 3 proprietary Chinese medicines, including Lianhuaqingwen granules, for the treatment of COVID-19 (Ni et al., 2020). ACE2 and TMPRSS2 proteins mediate SARS-CoV-2 virus invasion into host cells, and among all cancers, only patients with COAD and rectal adenocarcinoma showed upregulated expression of ACE2 and TMPRSS2 proteins *in vivo*, so patients with COAD and rectal adenocarcinoma may be more susceptible to contracting SARS-CoV-2 (Hoang et al., 2021; Wang and Yang, 2021). Furthermore, it was found that cancer patients infected with SARS-CoV-2 virus tend to have a poorer survival prognosis than healthy patients infected with SARS-CoV-2 virus due to the suppression of the systemic immune system caused by the effects of tumor and anti-cancer treatment (Liang et al., 2020). Based on the extensive biological functions of quercetin, including anti-tumor, anti-inflammatory and anti-viral, we used bioinformatics techniques and algorithms to work on the potential value and molecular mechanisms of quercetin for COAD/COVID-19.

In the current study, we constructed a COAD prognostic model with good predictive efficacy by 862 CCRG. 105 target genes of quercetin for COAD/COVID-19 were obtained by the network pharmacology approach. The PPI network revealed that the mechanism of quercetin for COAD/COVID-19 might be associated to apoptosis and oxidative stress. Apoptosis is a normal form of planned cell death, which occurs all the time in the human body (Fuchs and Steller, 2011). Once this orderly biological process is disrupted, too much or too little apoptosis can lead to disease. The inhibition of the normal apoptotic process, resulting in cell immortalization, is often one of the hallmark events of tumorigenesis. Dysregulation of apoptosis is closely associated not only with tumorigenesis and progression, but also with the emergence of tumor drug resistance (Pistritto et al., 2016). Through suppression of the PI3K/AKT/mTOR signaling pathway, SARS-CoV-2 spike pseudovirions promote the emergence of autophagy, which in turn leads to the onset of apoptosis (Li F. et al., 2021). Abnormal oxidative stress may lead to cancer, inflammation, diabetes, atherosclerosis and other diseases (Pisoschi and Pop, 2015). Dysregulation of oxidative stress is a risk factor for COAD. Quercetin effectively reverses oxidative stress in rats (the model of colon carcinogenesis) by mediating the NRF2/Keap1 pathway (Darband et al., 2020). Excessive oxidative stress reduces the function of the body’s immune system, leading to an increased likelihood of SARS-CoV-2 virus invasion of the body (Iddir et al., 2020). Based on the results of KEGG enrichment analysis, we found that the mechanism of quercetin for COAD/COVID-19 is closely associated to apoptosis, oxidative stress, anti-inflammatory, anti-viral, immune, anti-cancer and related pathways including IL-17, TNF, and HIF-1, which is generally consistent with the results of GeneMANIA analysis.

We identified FOS, NFKB1, JUNB, JUN, and NFKB1A as core target genes of quercetin for the treatment of COAD/COVID-19. The expression of c-Fos in COAD patients correlates with the occurrence of metastasis and clinical stage, and the specific molecular mechanism may be related to c-Fos upregulating the expression of genes related to epithelial mesenchymal transition, thus c-Fos may be a therapeutic target for patients with metastatic COAD (Ding et al., 2020). Recent studies have identified FOS as a potential drug target for COVID-19 patients through a bioinformatics approach (Ye et al., 2022). JUN has been implicated in tumorigenesis and progression, inducing the appearance of autophagy, apoptosis and cycle arrest in COAD cells (Vogt, 2001; Gao et al., 2018). The immune response of T cells is closely associated with recovery in COVID-19 patients, and these biological processes may be regulated by molecular mechanisms associated with JUN(Zheng et al., 2020). As an important paralog of JUN, JUNB is associated with the activation of the NF-κB pathway, which is participated in a number of key processes during tumorigenesis and progression (Hoesel and Schmid, 2013; Joung et al., 2022). NFKB1 and NFKB1A, subunits of NF-κB, are associated with cancer due to inflammation (Concetti and Wilson, 2018). NFKB1 deficiency induces the enhanced inflammatory response and the appearance of DNA damage in mice, which in turn leads to tumorigenesis (Cartwright et al., 2016). Since the NF-κB pathway is associated with increased cytokine storm and exacerbated complications of COVID-19, inhibitors of the NF-κB pathway may be used in the clinical treatment of patients with COVID-19 (Hariharan et al., 2021).

## Conclusion

In this study, we constructed a prognosis model for COAD patients by using CCRG and explored the targets and therapeutic mechanisms of quercetin for the treatment of COAD/COVID-19 comorbidity through bioinformatics analysis. We found that the therapeutic mechanisms may be related to oxidative stress, apoptosis, anti-inflammatory, immune, anti-viral and multiple pathways containing IL-17, TNF, HIF-1. The results of this study may provide new directions for basic experimental research and clinical treatment of COAD/COVID-19. However, the therapeutic effects of quercetin have not been validated in clinical trials, so we still need to conduct clinical trials in subsequent studies to clarify its therapeutic value.

## Data Availability

The datasets presented in this study can be found in online repositories. The names of the repository/repositories and accession number(s) can be found in the article/Supplementary Material.

## Abbreviations

AUC, under the curve; BP, biological processes; CC, cellular components; COAD, colon adenocarcinoma; CCRG, COAD/COVID-19 related genes; C-index, concordance index; DEG, differentially expressed genes; GEO, Gene Expression Omnibus; GO, Gene ontology; KEGG, Kyoto Encyclopedia of Genes and Genomes; KM, Kaplan–Meier; LASSO, least absolute shrinkage and selection operator; MF, molecular functions; PPI, protein-protein interaction; RG, related genes; ROC, receiver operating characteristic; TCGA, The Cancer Genome Atlas
